# Genetic Characterization of West Nile Virus Lineage 2, Greece, 2010

**DOI:** 10.3201/eid1705.101759

**Published:** 2011-05

**Authors:** Anna Papa, Tamás Bakonyi, Kyriaki Xanthopoulou, Ana Vázquez, Antonio Tenorio, Norbert Nowotny

**Affiliations:** Author affiliations: Aristotle University, Thessaloniki, Greece (A. Papa, K. Xanthopoulou);; Faculty of Veterinary Science, Budapest, Hungary (T. Bakonyi);; University of Veterinary Medicine, Vienna, Austria (T. Bakonyi, N. Nowotny);; Instituto de Salud Carlos III, Majadahonda, Spain (A. Vázquez, A. Tenorio)

**Keywords:** viruses, West Nile virus, lineage 2, flavivirus, neuroinvasive, human, mosquito, Greece, dispatch

## Abstract

We conducted a complete genome analysis of a West Nile virus detected in *Culex pipiens* mosquitoes during a severe outbreak of human West Nile disease in Greece 2010. The virus showed closest genetic relationship to the lineage 2 strain that emerged in Hungary in 2004; increased virulence may be associated with amino acid substitution H249P.

West Nile virus (WNV) is a flavivirus maintained in an enzootic cycle between bird amplifying hosts and ornithophilic mosquito vectors, mainly *Culex* species; humans, horses, and other mammals are incidental hosts. Although most human WNV infections remain subclinical, febrile illness develops in ≈20% of infected persons and neuroinvasive disease in <1%. Severe disease is more frequent among the elderly and immunocompromised (*1*).

WNV strains are classified into at least 7 putative genetic lineages (*2*). Lineage 1 strains are the most widespread, found in Africa, Europe, Asia, Australasia (“Kunjin virus”), and America, while lineage 2 strains are mainly distributed in sub-Saharan Africa and Madagascar. WNV of proposed lineage 3 (“Rabensburg virus”) is circulating in certain *Culex* and *Aedes* species mosquitoes in southern Moravia, Czech Republic, close to the Austrian border, without recognized pathogenicity for mammals (*3*). Strain LEIV-Krns88-190, isolated from *Dermacentor marginatus* ticks from the Caucasus represents proposed lineage 4 of WNV. A new lineage, lineage 5, has been proposed for Indian isolates previously comprising lineage 1c, and a reclassification as lineage 6 has been proposed for the Sarawak Kunjin virus strain, which is markedly different from the other Kunjin viruses. Furthermore, a seventh lineage has been suggested for the African Koutango virus, which is closely related to WNV (*2*), and an eighth lineage has been proposed on the basis of WNV sequences detected in *Cx. pipiens* mosquitoes captured in southern Spain in summer 2006 (*4*).

WNV strains differ considerably in virulence and neuroinvasiveness. Since neuroinvasive isolates mainly belonged to lineage 1, lineage 2 strains were previously considered to be less virulent. Recent studies, however, indicate that several highly virulent and neuroinvasive strains of lineage 2 WNV were detected in southern Africa (*5*).

Until 2004, only lineage 1 and 3 WNV strains had been found in Europe. A lineage 2 strain emerged in 2004 in Hungary in birds of prey (*6*), which established itself in the region and exhibited explosive geographic spread in 2008 throughout Hungary and into eastern Austria. Besides deaths in birds and domesticated mammals, human neurologic WNV cases have been diagnosed in the affected regions during the epidemic seasons since 2004. However, the human cases of WNV neuroinvasive disease have been comparatively rare and rather mild with no deaths.

In Greece, where WNV cases had not previously been reported, a 2007 study, conducted in an area near a delta where 4 rivers enter the Aegean Sea, 4 (1%) of 392 persons exhibited neutralizing WNV antibodies, 2 with high titers. These findings suggested that WNV, or an antigenically closely related flavivirus, circulates, at least locally, in rural areas in Greece (*7*). This area was the focus of a large WNV outbreak in summer–autumn 2010 (*8*). Up to October 4, 2010, a total of 191 neuroinvasive human cases have been laboratory diagnosed, with 32 deaths, all in elderly patients.

Soon after the first human cases were recognized, mosquitoes were trapped at the sites where the cases occurred and tested for WNV. One pool, consisting of 50 *Cx. pipiens* mosquitoes, trapped during the night of August 10, 2010, in Nea Santa (40.84194°N, 22.91499°E), a village 30 km northeast of Thessaloniki, was found to be positive for WNV RNA by reverse transcription–PCR. Sequencing of a 146-nt fragment of the nonstructural protein 5 (NS5) gene gave the first evidence that the virus belongs to lineage 2, and that it is highly similar to the strain that emerged in Hungary in 2004 and to isolates from fatal human and animal cases in South Africa (*9*). The purpose of this study was to establish the complete genomic sequence of the WNV identified in a pool of mosquitoes trapped at the site of an ongoing West Nile disease epidemic in humans, and investigate it for potential (neuro)virulence/pathogenicity markers.

## The Study

To amplify overlapping PCR products of the WNV from the Greek outbreak, we used RNA from the above-mentioned mosquito pool and reverse transcription–PCRs designed for the amplification of the complete genome of lineage 2 WNV, which had emerged in Hungary in 2004 (strain goshawk-Hungary-2004, GenBank accession no. DQ116961; *6*). Amplification products were directly sequenced in both directions; sequences were aligned and compiled, and an 11,028-nt continuous sequence was obtained, representing the nearly complete genome sequence of the virus, designated Nea Santa-Greece-2010. Because of the limited amount of sample material, the last 20 nt of the 5′ and 3′ ends were not determined by rapid amplification of cDNA ends. The nucleotide sequence was deposited in GenBank database under accession no. HQ537483.

The nucleotide sequence of the putative open reading frame 1 (starting at nt position 97) was translated to a 3,434-aa polypeptide sequence. The nucleotide and putative amino acid sequences were aligned with all complete lineage 2 WNV nt and aa sequences available in GenBank, as well as with representatives of other WNV lineages.

The highest (99.6%) nucleotide identity of the Nea Santa-Greece-2010 sequence was found to the goshawk-Hungary-2004 strain. Only single nucleotide substitutions (n = 44) were detected, equally distributed over the genome. The putative aa sequences of the polyprotein precursor of the 2 viruses were 99.7% identical. The alignment of the putative precursor polypeptide sequences showed aa substitutions unique for the European lineage 2 WNVs. These substitutions were found in the nonstructural proteins (Table).

**Table:** Unique amino acid substitutions in the putative nonstructural proteins of the Nea Santa-Greece-2010 lineage 2 West Nile virus*

Strain	Protein, aa position														
	NS1		NS2B			NS3			NS4B						NS5
	44		88	119		11	249		14	23	32	49	113		190
Gr-10 (lin. 2)	Arg		Ile	Ile		Arg	**Pro**		Gly	Thr	Asn	Ala	Met		Arg
Hu-04 (lin. 2)	Arg		Ile	Val		Arg	His		Ser	Thr	Asn	Thr	Val		Lys
Other lin. 2 WNV strains	Lys		Met	Val		Lys	His		Ser†	Ala	Ser	Thr‡	Val		Lys
NY-99 (lin. 1)	Lys		Met	Val		Lys	**Pro**		Ser	Val	Glu	Thr	Val		Lys
Rab-97 (lin. 3)	Arg		Ile	Val		Lys	Asn		Ser	Thr	Asp	Ser	Val		Lys
Rus-98 (lin. 4)	Lys		Met	Val		Arg	Thr		Gly	Ser	Ser	Ser	Val		Lys
Ind-80 (lin. 5)	Lys		Met	Val		Lys	Thr		Gly	Ala	Glu	Thr	Val		Lys

*NS, nonstructural protein; aa, amino acid; lin., lineage; Arg, arginine; Ile, isoleucine; Pro, proline; Gly, glycine; Thr, threonine; Asn, asparagine; Ala, alanine; Met, methionine; Val, valine; His, histidine; Lys, lysine; Ser, serine; WNV, West Nile virus. Abbreviations of strains are explained in the Figure legend. **Boldface** indicates the His→Pro substitution at aa position 249. †Except SE-90, WNFCG, Mad-78, and Sarafend (Gly). ‡Except Mad-78 (Ala).

Several studies have focused on the determination of genetic markers for pathogenicity and virulence of WNV strains. In the New York strain of WNV, envelope protein glycosylation proved to be a molecular determinant of neuroinvasiveness (*10*). The A_30_P substitution in the NS2A protein reduced the ability of the virus to inhibit interferon induction (*11*). The introduction of a T_249_P amino acid substitution in the NS3 helicase in a low-virulence strain was sufficient to generate a phenotype highly virulent to American crows (*12*). The C_102_S mutation in the NS4A protein also influenced virulence and thermosensitivity in a mouse model (*13*). An adaptive mutation E_249_G in the same protein reduced virus replication in mouse cells (*14*). In lineage 2 strains, substitutions in the NS3 protein (S_160_A and R_298_G), NS4A protein (A_79_T), and NS5 protein (T_614_P, M_625_R, and M_626_R) were predicted to be possible virulence markers (*15*). All these foci were checked in the Nea Santa-Greece-2010 strain, but differences were not found, except for the 249 residue of the NS3 protein. All investigated lineage 2 viruses (including goshawk-Hungary-2004) contain histidine at this locus, while the Greek sequence contains proline, similar to several neuroinvasive lineage-1 WNV strains. Previous experimental studies have attributed this mutation to a higher capacity of the virus to replicate in corvids (*12*), which likely would result in increased virus transmission rates.

Viruses of lineages 3, 4, and 5 contain aspargine, threonine, and threonine at this locus, respectively (Table). The inferred phylogenetic relationships between the investigated complete genome nucleotide sequences are delineated in a phylogram (Figure). The Nea Santa-Greece-2010 and goshawk-Hungary-2004 viruses form a close, monophyletic group that clusters with neurovirulent lineage 2 strains isolated in South Africa.

**Figure:**
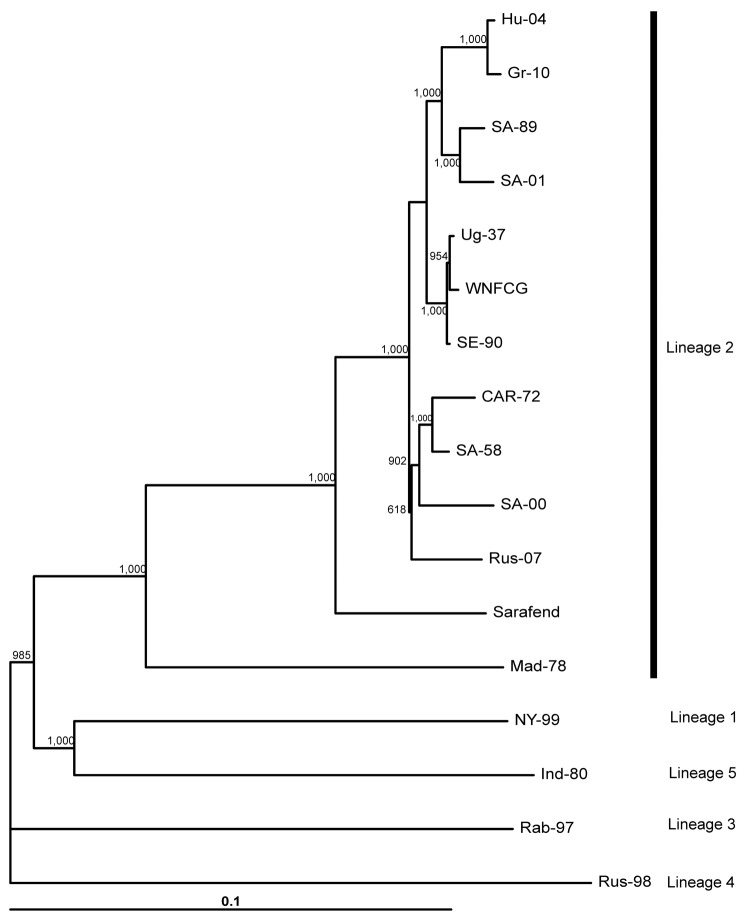
Neighbor-joining phylogram based on complete genome nucleotide sequences of selected West Nile virus strains. Strain abbreviations (isolation source, country, year, GenBank accession no.): Hu-04: *Accipiter gentilis*, Hungary, 2004, DQ116961; Gr-10: *Culex pipiens*, Greece, 2010, HQ537483; SA-89: human, South Africa, 1989, EF429197; SA-01: human, South Africa, 2001, EF429198; Ug-37: human, Uganda, 1937, AY532665; WNFCG: derivate of Ug-37, M12294; SE-90: *Mimomyia lacustris*, Senegal, 1990, DQ318019; CAR-72: *Cx. tigripes*, Central African Republic, 1972, DQ318020; SA-58: human, South Africa, 1958, EF429200; SA-00: human, South Africa, 2000, EF429199; Rus-07: human, Russia, 2007, FJ425721; Sarafend: derivate of Ug-37, AY688948; Mad-78: *Coracopsis vasa*, Madagascar, 1978, DQ176636; NY-99: human, USA, 1999, AF202541; Ind-80: human, India, 1980, DQ256476; Rab-97: *Cx. pipiens*, Czech Republic, 1997, AY765264; Rus-98: *Dermacentor marginatus*, Russia, 1998, AY277251. Rus-98 was used as outgroup. Bootstrap values of 1,000 replicates are shown. The main genetic lineages are indicated on the right. Horizontal bar shows genetic distance.

## Conclusions

Epidemiologic observations of the lineage 2 WNV in Europe during 2004–2010, and the close genetic relatedness of the WNV circulating in Hungary and eastern Austria to the virus identified in *Cx. pipiens* mosquitoes during the 2010 outbreak in Greece, indicate that, most likely, descendants of the goshawk-Hungary-2004 strain spread southward to the Balkan Peninsula and reached northern Greece. Our data indicate that an independent introduction of a highly similar lineage 2 WNV from Africa to northern Greece is unlikely. The importance of the H_249_P aa change in the NS3 protein of the Nea Santa-Greece-2010 virus should be verified in experimental studies. This aa change also emphasizes the value of close genetic monitoring of strains involved in additional European WNV outbreaks.
